# Rapid Analysis of Pacific Ciguatoxins in Fish Extracts with a Lateral Flow Assay

**DOI:** 10.1021/acs.analchem.5c06159

**Published:** 2026-01-09

**Authors:** Ulises G. Díaz-Avello, Vasso Skouridou, Takeshi Tsumuraya, Masahiro Hirama, Naomasa Oshiro, Mònica Campàs, Ciara K. O’Sullivan

**Affiliations:** † Interfibio Research Group, Departament d’Enginyeria Química, 16777 Universitat Rovira i Virgili, 43007 Tarragona, Spain; ‡ Institute of Agrifood Research and Technology (IRTA), 43540 La Ràpita, Spain; § Department of Biological Chemistry, Graduate School of Science, 639061 Osaka Metropolitan University, Osaka 599-8570, Japan; ∥ Division of Biomedical Food Research, 243142 National Institute of Health Sciences, Kanagawa 210-9501, Japan; ⊥ Institució Català de Recerca i Estudis Avancats (ICREA), 08010 Barcelona, Spain

## Abstract

The increasing spread of toxin-producing *Gambierdiscus* dinoflagellates has led to cases of ciguatera poisoning in previously nonendemic regions. Ciguatoxins (CTXs) are potent, heat-stable compounds responsible for ciguatera, and their rapid and reliable analysis in seafood is essential to prevent illness. This study presents the development of the first lateral flow assay (LFA) for the detection of major Pacific CTXs congeners. The assay employs previously developed monoclonal antibodies in a sandwich format: specific antibodies against CTX1B and CTX3C are immobilized on a single test line, while the reporter antibody is a single antibody recognizing major Pacific CTX congeners that is conjugated to gold nanoparticles. The LFA provides one-step visual results in 30 min, making it suitable for resource-limited settings. It exhibits a visual limit of detection of 400 pg/mL and the assay is highly specific, showing no cross-reactivity with other marine toxins. It successfully detected Pacific CTXs spiked in noncontaminated fish extracts, as well as in naturally contaminated samples as previously confirmed by LC-MS/MS, with an estimated cutoff of approximately 0.1 μg/kg. Overall, this LFA demonstrates great potential as a rapid screening tool for outbreak investigations and research programs.

## Graphical abstract

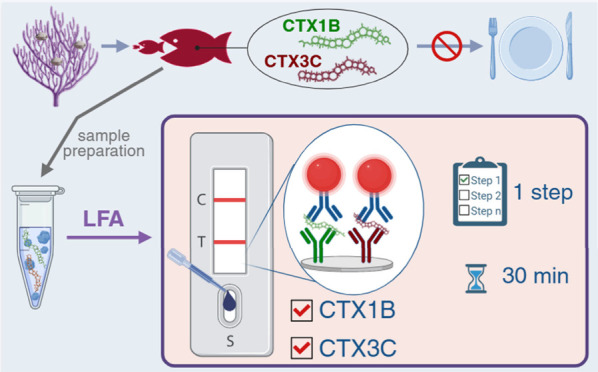

Marine toxins are naturally occurring compounds primarily produced by certain types of algae, cyanobacteria, diatoms, and dinoflagellates.^1^ They can cause mild to severe illness in humans following the ingestion of contaminated seafood, presenting a wide spectrum of gastrointestinal and neurological symptoms.^2^ Ciguatoxins (CTXs) are potent neurotoxins responsible for ciguatera poisoning (CP), the most frequent seafood-borne illness worldwide,^3^ and they or their precursors are produced by benthic dinoflagellate species of *Gambierdiscus*.^4^ Ciguatera can cause a variety of gastrointestinal, neurological, and cardiovascular symptoms, often complicating diagnosis due to their nonspecific nature.^5^ It is estimated to affect up to 50,000 people annually, although challenges related to accurate diagnosis and the lack of accessible detection tools are believed to result in significant under-reporting.^6^ Ciguatera was traditionally associated with tropical and subtropical regions; however, the rising water temperatures, disturbance of coral reefs and the globalization of the seafood trade may have contributed to its expansion to nonendemic areas.^3,4,7,8^ It is now considered an emerging risk in Europe due to the reported intoxication cases, as well as the presence of *Gambierdiscus* in coastal areas and the detection of CTXs in fishes.^6,9,10^

Ciguatoxins are lipid-soluble polycyclic ether compounds with more than 30 congeners, which can be classified into different groups based on their chemical structure.^3,4^ Pacific CTXs are generally considered as the most potent ones, exhibiting high intraperitoneal toxicity in mice, with half-lethal-dose (LD_50_) values of 0.25–0.9 μg/kg of body weight.^6^ CTX1B (known as P-CTX-1), in particular, is one of the major congeners in the Pacific and the most potent one among its regional analogues, and it has been extensively characterized since the 1990s.^6,11^ The United States Food and Drug Administration (FDA) has established guidance levels of 0.01 μg/kg CTX1B equivalents and 0.1 μg/kg Caribbean CTX-1 (C-CTX1).^7^ There is no explicit regulatory limit in Europe, but it is prohibited to sell fish with detectable levels of CTXs.^6^ CTXs are heat-stable, cannot be eliminated by cooking or freezing, and have no known antidotes. Consequently, the most effective strategy to mitigate the risk of CP is to avoid consumption of contaminated seafood, which relies on early and reliable detection of the toxins.

Different methods exist for the detection of CTXs which can be broadly classified into biological, chemical and biochemical.^12–14^ Biological methods include the mouse bioassay, cell-based cytotoxicity assays,^15^ and receptor binding assays.^16^ While they offer several advantages, limitations in sensitivity and specificity, costs, and ethical concerns have driven the development of alternative assays. Chemical methods, and specifically liquid chromatography-tandem mass spectroscopy (LC-MS/MS), are the leading techniques for not only detecting CTXs but also providing toxin profiles, and they exhibit very high sensitivity and specificity.^17^ Biochemical assays rely on the use of specific antibodies that recognize CTXs in Enzyme-Linked Immunosorbent Assays (ELISA) and different biosensor formats.

The efforts in developing CTX-specific antibodies began in the late 1970s,^18^ but it was not until the beginning of the 2000s that high-affinity and specific antibodies against Pacific CTXs were reported. Using rationally designed synthetic haptens mimicking parts of the CTXs molecules, mouse monoclonal antibodies were developed against the left wings of CTX1B (3G8 IgG)^19^ and CTX3C (10C9 IgG),^20^ and an antibody against the right wing of CTX1B, also able to bind to the right wing of CTX3C, even though it lacks the M-ring hydroxyl group (8H4 IgG).^21^ By combining the three antibodies 3G8, 10C9, and 8H4 in one single sandwich ELISA, they demonstrated the detection of any of the four main congeners of Pacific CTXs (CTX1B, 54-deoxyCTX1B, CTX3C and 51-OH-CTX3C) with a limit of detection (LOD) of less than 1 pg/mL and the successful detection of CTX1B spiked in fish flesh at the FDA guidance level of 0.01 μg/kg.^22^ The same antibodies were later exploited for the development of magnetic bead-based colorimetric assays and electrochemical biosensors with LODs at the low pg/mL level.^23–26^

Lateral flow assays (LFA) have garnered a lot of interest over the last years for the detection of marine toxins.^27,28^ These simple, rapid, and low-cost tests can be used in resource-limited settings and can provide visual results in less than 30 min, facilitating timely decision-making and ensuring food safety. The majority of LFAs reported in the literature to date have employed antibodies in a competitive format with signal-off colorimetric visual detection. They targeted primarily tetrodotoxin (TTX), as well as okadaic acid (OA), domoic acid (DA), saxitoxins (STXs), dinophysistoxins (DTXs), brevetoxin B (PbTx-2) and cyclic imines (CIs), for single analyte or multiplex detection of several toxins simultaneously (Supporting Information Table S1).

In this work, we report the first LFA test for the detection of the main congeners of CTX1B and CTX3C with one single test, based on a signal-on sandwich format employing the antibodies previously developed by Tsumuraya and co-workers.^29^ The design of the LFA test is illustrated in Figure 1a: the 3G8 IgG against the left wing of CTX1B and the 10C9 IgG against the left wing of CTX3C are immobilized at equal concentrations at the test line, whereas the 8H4 IgG against the common right wing of CTX1B and CTX3C is conjugated to 40 nm gold nanoparticles (8H4@AuNPs conjugate) and deposited on the conjugate pad. Once the sample is applied to the sample pad, it flows through the strip by capillary forces. If any of the main congeners of CTXs is present in the sample (CTX1B, 54-deoxyCTX1B, CTX3C or 51-OH-CTX3C), it will be bound by the 8H4 antibody immobilized on the AuNPs and the complex will be captured at the test line by one of the two antibodies, depending on the specific CTX present in the sample. This will ultimately result in the formation of a red line. Remaining 8H4@AuNPs conjugate will be captured at the control line by an anti-mouse antibody, forming a red line and indicating correct function of the test (Figure S1).

**Figure 1:**
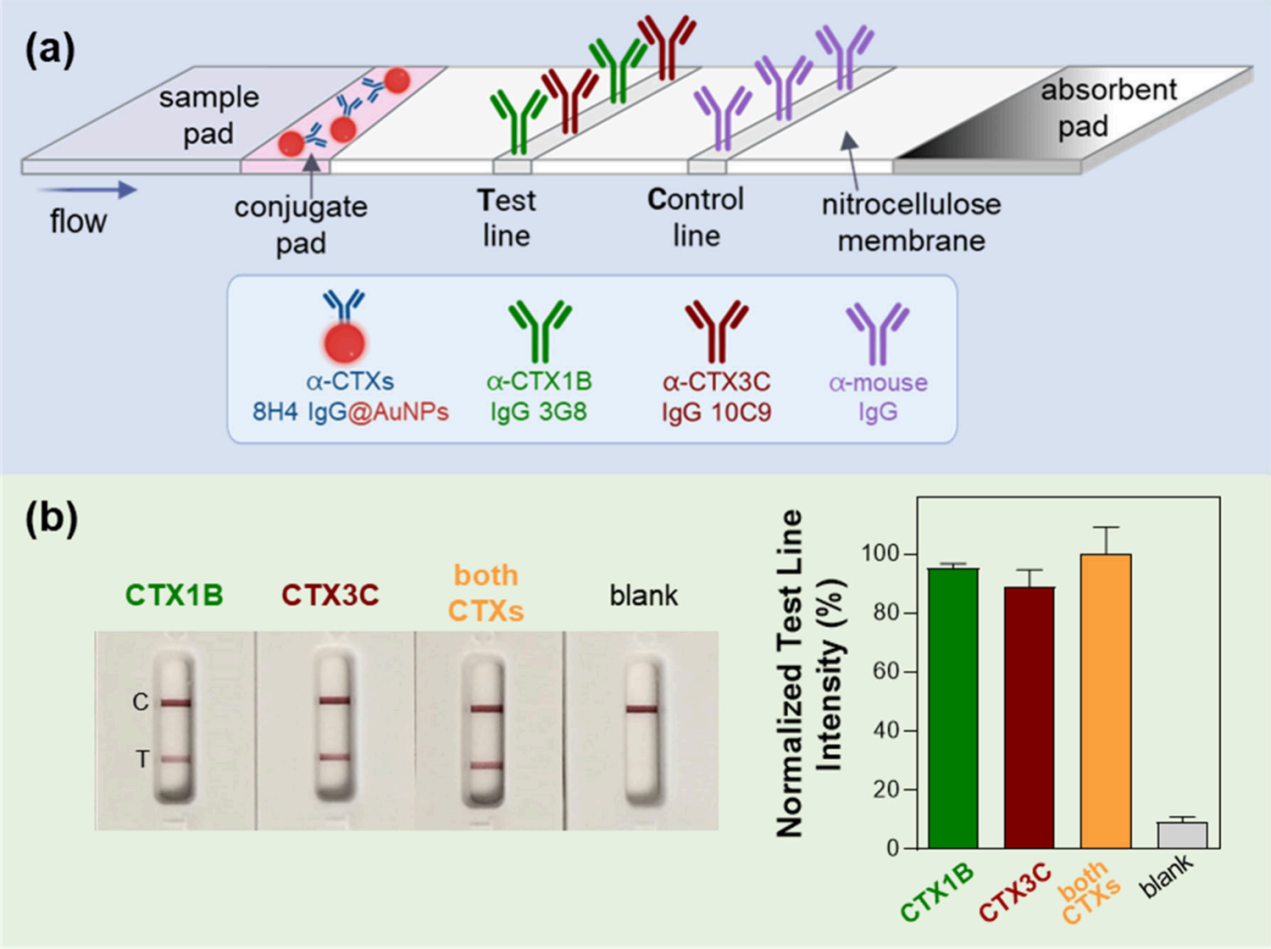
Lateral flow assay (LFA) test for the detection of the main congeners of Pacific ciguatoxins (CTXs). (a) Design of the test. (b) Detection of the CTX1B and 51-OH-CTX3C, individually (1000 pg/mL each) and combined (500 pg/mL each), with the LFA test. Test line intensities were quantified with the Cube LFA reader (*n* = 2, error bars: ± SD) and were normalized to the % of highest mean signal in the data set.

The 8H4@AuNPs conjugate was prepared by first optimizing the concentration of the antibody required to provide colloidal stability (Figure S2a), and the final conjugate was characterized by UV–vis spectroscopy (Figure S2b). The tests were prepared and assembled as detailed in the Supporting Information. The design of the test was evaluated by analyzing CTX1B and 51-OH-CTX3C individually and in combination. Due to the lack of high-quality and accurately quantified CTX3C standards, the 51-OH-CTX3C congener was used as a representative model for CTX3C-type congeners, since it is recognized by the corresponding antibodies to the same extent as CTX3C.^21,22^ Each sample (50 μL) was applied to the LFA test, followed by the addition of running buffer (100 μL). Test line intensities were measured after 30 min with a portable LFA reader. As shown in Figure 1b, the relative intensities obtained for each individual toxin at 1000 pg/mL were comparable to that of the mixture containing both toxins at 500 pg/mL each (total concentration of 1000 pg/mL). These results confirm that the test is capable of detecting both toxins simultaneously without signal interference.

Several test parameters were subsequently optimized to enable the sensitive detection of CTXs. First, LFAs incorporating different nanoparticle conjugates were fabricated to identify the one providing the most sensitive and specific detection. To this end, the reporter 8H4 antibody was conjugated to (a) spherical AuNPs (40 nm), (b) gold nanourchins (AuNUs; 90 nm), (c) streptavidin immobilized on AuNPs (SA@AuNPs; 40 nm) and (d) carbon nanoparticles (CNPs; 25 nm average primary particle size) (Supporting Information). AuNUs are gold nanoparticles with spiky and uneven surfaces, exhibiting unique plasmonic properties with increased surface area compared to spherical AuNPs.^30^ SA@AuNPs are often combined with biotinylated antibodies to achieve higher loading and potentially improve orientation and sensitivity.^31^ Finally, CNPs are small, black, amorphous nanoparticles that can be efficiently functionalized with proteins. They are considerably more cost-effective than AuNPs and provide high visual contrast on white membranes.^32^ CTX1B was analyzed at varying concentrations with each LFA, and the highest signal and signal-to-noise ratio were observed with spherical 40 nm AuNPs (Figure S3). Therefore, this conjugate was selected for further test development. The effect of nitrocellulose (NC) membrane type on signal intensity and assay run time was then evaluated. Two commonly used NC membranes, FF120HP and FF170HP, were compared, with FF170HP having smaller pores and a slower flow rate. When FF170HP was used, higher test line intensities were observed within a shorter assay time (25–30 min) as compared to FF120HP (Figure S4), and it was therefore selected for further development. The concentrations of antibodies used for constructing the test line were subsequently optimized. In parallel, the potential application of a silver enhancement step was assessed, based on previous studies demonstrating its effectiveness in improving assay sensitivity.^33^ The highest test line intensity was observed when 1.5 mg/mL of each antibody (3G8 and 10C9) was used instead of 1 mg/mL (Figure S5). A slight increase in the signal was observed for an assay run time longer than 20 min, while silver enhancement had no significant effect. Therefore, a concentration of 1.5 mg/mL for both antibodies and an assay time of 30 min without silver enhancement were selected as the final test conditions.

All previous optimizations were performed using unblocked NC membranes, the sample pads were pretreated with a blocking agent (1% w/v BSA) and a detergent (0.05% w/v Tween-20), and the LFA running buffer was PBS. Blocking agents, often used in combination with detergents, are typically employed to prevent nonspecific signals and improve sensitivity by increasing the signal-to-noise ratio. To simplify test fabrication, unblocked membranes are preferred and any blocking agents required are incorporated in the sample pad.^34^ In the case of the CTXs LFA test, nonspecific signals were occasionally observed when unblocked membranes were used in combination with pretreated sample pads. To eliminate these nonspecific signals, several running buffer additives were evaluated. BSA and skimmed milk resulted in the lowest test line intensities in blank samples without CTXs, although the lines were still visible to the naked eye, as any intensity above 10–12 arbitrary units (a.u., per LFA reader) is discernible (Figure S6a). The addition of BSA (1% w/v) to the running buffer was then assessed using both unblocked and blocked NC membranes. Blocking the membranes effectively eliminated nonspecific signals, and supplementation of the running buffer with BSA was not necessary when blocked membranes were used (Figure S6b). However, to prevent nonspecific signals when analyzing complex samples such as fish extracts, blocked membranes and BSA-supplemented running buffer were used.

The analytical sensitivity of the optimized LFA test was finally evaluated by constructing calibration curves for CTX1B and 51-OH-CTX3C separately. As shown in Figure 2a, test lines with gradually increasing intensities were observed with increasing CTXs concentration, consistent with signal-on tests. The visual limits of detection (vLOD) were 400 pg/mL for both CTX1B and 51-OH-CTX3C. When the portable LFA reader was used to measure the test line intensities, the LODs calculated from the sigmoidal curves were 306 pg/mL for CTX1B and 426 pg/mL for 51-OH-CTX3C. To further demonstrate the sensitivity of the assay, we tested each CTX individually at a concentration below the LOD, as well as a mixture in which the combined concentration corresponded to the vLOD. While the individual toxins at 200 pg/mL produced no visible test lines, their 200 + 200 pg/mL mixture resulted in a detectable positive signal (Figure S7). Overall, the vLOD of this test is superior to that of most LFA tests reported to date for other marine toxins (Table S1), potentially due to the sandwich format of the assay as compared to the competitive format typically employed for small molecules, as well as the high affinity of the monoclonal antibodies.

**Figure 2:**
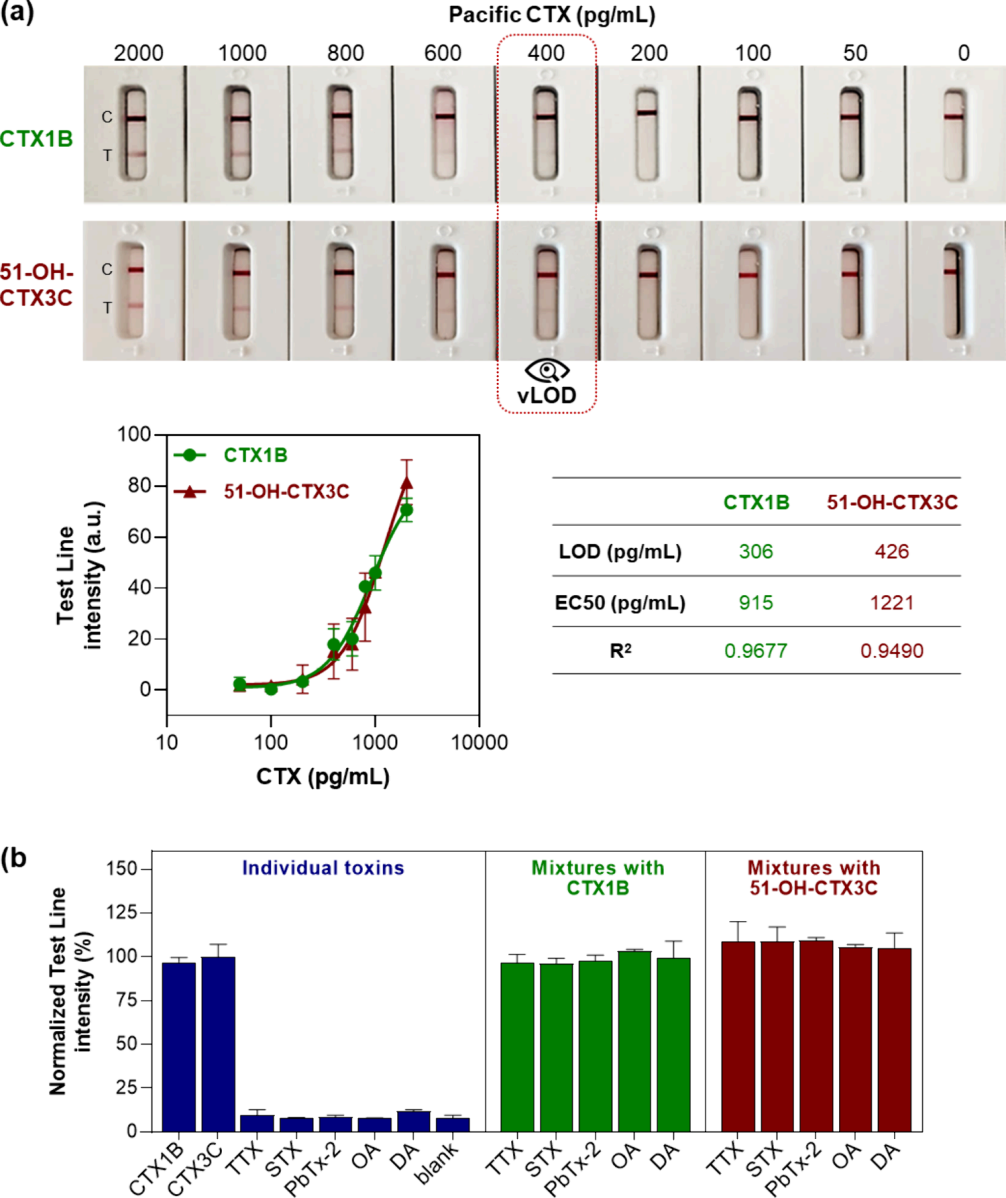
Sensitivity and specificity of the lateral flow assay (LFA) tests for the detection of CTX1B and 51-OH-CTX3C. (a) Calibration curves and determination of the limit of detection (LOD) in the buffer. Test line intensities were measured with the CubePlus LFA reader (*n* = 2, error bars: ± SD). (b) Specificity of the LFA test. All toxins were tested at 1000 pg/mL, individually or in mixtures as indicated (*n* = 2, error bars: ± SD). Where indicated, test line intensities measured with the LFA reader were normalized to the % of highest mean signal in the data set. All sample volumes were 50 μL. TTX: tetrodotoxin; STX: saxitoxin; PbTx-2: brevetoxin B; OA: okadaic acid; DA: domoic acid.

Furthermore, the test demonstrated high specificity, as no signals were observed for the other marine toxins tested (saxitoxin, domoic acid, okadaic acid, tetrodotoxin, and brevetoxin B) (Figure 2b). The presence of these toxins in the same sample did not interfere with the detection of CTXs, as indicated by the comparable relative signal intensities observed for CTXs tested alone and in combination with the other marine toxins.

The suitability of the Pacific CTXs LFA test for analyzing fish samples was assessed by using noncontaminated and contaminated extracts, prepared as detailed in the Supporting Information. First, an extract from noncontaminated *Lutjanus bohar* was spiked with CTX1B (50 μL of 600 pg/mL) at various matrix concentrations. Initial tests were conducted using unblocked NC membranes. CTX1B was successfully detected in all spiked extracts, regardless of matrix concentration, but significant overestimation was observed, with recoveries ranging from 161% to 178% (Figure 3a). In addition, nonspecific signals were detected with the neat (nonspiked) extracts. These background signals were effectively eliminated by using LFA strips fabricated with blocked NC membranes. When undiluted fish extract (50 μL of 5000 mg flesh equiv./mL, corresponding to 250 mg flesh equiv.) was used, both CTX1B and 51-OH-CTX3C, spiked in the extract at 1000 pg/mL, were efficiently detected with improved accuracy, resulting in recoveries of 115.3% and 113.7%, respectively (Figure 3b).

**Figure 3:**
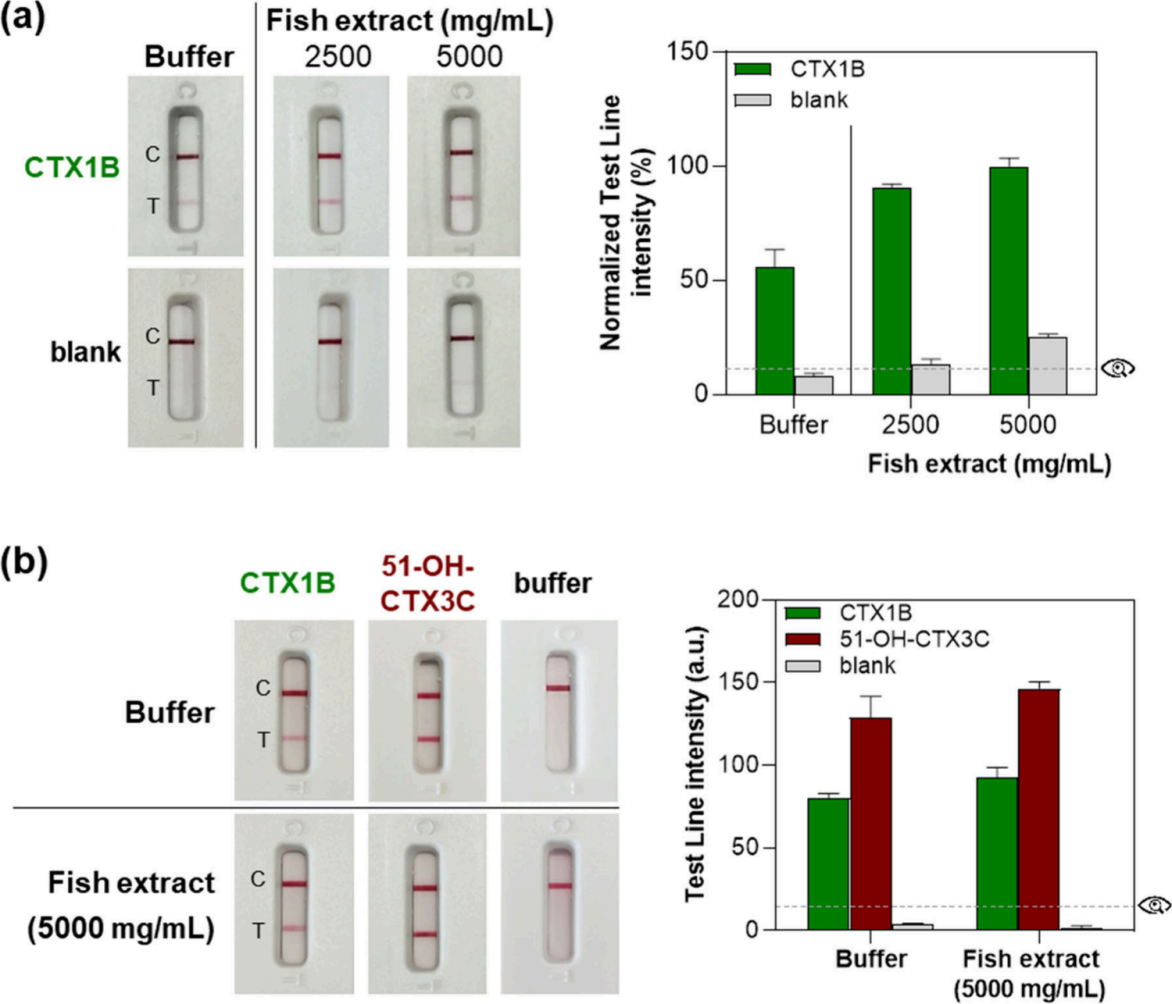
Detection of spiked CTX1B and 51-OH-CTX3C in noncontaminated *Lutjanus bohar* fish extract with the lateral flow assay (LFA) tests. (a) CTX1B (600 pg/mL) was spiked in buffer (PBS) or in fish extract at 2500 or 5000 mg flesh equiv./mL and analyzed using tests with unblocked membranes. (b) CTXs (1000 pg/mL) were spiked in 5000 mg flesh equiv./mL fish extract and analyzed using LFA tests with blocked membranes. In all cases, 50 μL of test sample was applied to each strip. Test line intensities were quantified with the CubePlus LFA reader (*n* = 2, error bars: ± SD) and normalized to the % of highest mean signal in the data set. The horizontal dashed line and the eye icon indicate the visual limit of detection, below which no signal could be observed by naked eye inspection.

The LFA test was finally applied to naturally contaminated fish extracts from *L. bohar*, *L. monostigma* and *Variola louti* specimens (Table S2 and Figure 4). All extracts were analyzed without dilution, with the exception of samples FE20111 and FE20112, which were diluted 3-fold and 2-fold, respectively, prior to analysis. Out of the ten samples analyzed, nine had been previously confirmed to contain CTXs by LC-MS/MS,^25^ while one was negative (Table S2). The LFA test detected CTXs in seven of the nine positive samples (Figure 4). False-negative results were observed for two samples: FE24002 and FE24003. Sample FE24003 contained a very low concentration of CTXs (66 pg/mL), below the LFA’s LOD (300–400 pg/mL), hence explaining the negative result. The false-negative result for sample FE24002 by visual inspection remains unclear, considering that LC-MS/MS reported a concentration of 543 pg/mL. It should be noted that CTX1B was the least abundant congener and that the 52-*epi*-54-deoxyCTX1B is probably less well recognized by the assay. Nevertheless, this sample was correctly identified as CTX-positive when the LFA reader was used to measure the intensity of the test line. The LFA test also correctly identified the negative sample as CTX-free.

**Figure 4:**
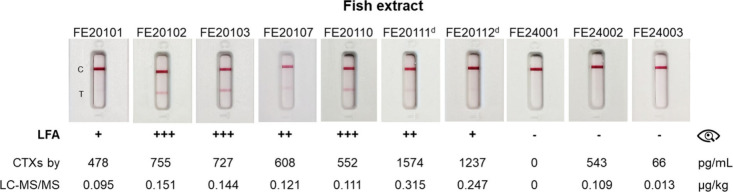
Analysis of fish extracts with lateral flow assay (LFA) tests. The extracts were analyzed directly at 5000 mg flesh equiv./mL (without dilution), except for # NIHS-FE20111 (1667 mg flesh equiv./mL; dilution 1/3) and # NIHS-FE20112 (2500 mg flesh equiv./mL; dilution 1/2), annotated with “^d^”. The total CTXs were quantified by LC-MS/MS at 5000 mg flesh equiv./mL. The LFA results by visual inspection were scored as negative (−), weak positive (+), positive (++) and strong positive (+++). Each sample was analyzed in duplicate.

It is necessary to mention that, in the present study, the naturally incurred samples were prepared following a multistep extraction and purification protocol, which produced highly purified fish extracts.^25^ This, together with the use of blocked NC membranes, certainly contributed to the good performance observed when applying the LFA to both spiked and naturally contaminated samples, as cleaner extracts reduce potential interferences from matrix components. It is also important to note that matrix effects cannot be fully predicted, as the composition of fish tissues varies considerably between species, individuals, size and origin.^14^ This variability may influence assay performance and could also explain, at least in part, the few inconsistent results. While the extraction protocol used here is effective, it is also relatively labor-intensive. There may be room for simplification of the extraction procedure to make the assay more suitable for field deployment. Future research should therefore focus on the development of rapid, reliable, and efficient extraction methods that ensure high recoveries of CTXs while maintaining minimal matrix interference. Optimizing sample processing and toxin extraction workflows will be essential to fully assess and expand the applicability of this LFA to field settings.

In conclusion, a simple and rapid LFA test was developed for the detection of the main congeners of Pacific CTXs. The test enables simple, one-step visual screening of fish samples within 30 min and operates with a cutoff of approximately 0.1 μg/kg, as demonstrated by the analysis of samples FE20101 and FE20110 without prior dilution. This cutoff is sufficient to detect CTXs in the food remnants of CP patients, which have been reported to contain a minimum of 0.025 MU/g (equivalent to 0.175 μg/kg CTX1B).^35^ Lehane and Lewis^36^ also reported that CTX1B can induce ciguatera symptoms at concentrations as low as 0.1 μg/kg. Other studies have similarly suggested that a threshold of 0.1–0.2 μg/kg could be more pragmatic for CP risk management than the U.S. FDA’s cautionary guidance level of 0.01 μg/kg.^37,38^ Achieving such sensitivity remains challenging due to the limited availability and high cost of purified CTX standards, which constrain the development of more sensitive detection methods.

## Supplementary Material



## Notes

Materials and reagents; Fish samples and preparation of extracts; Preparation of antibody-nanoparticle conjugates; Preparation and assembly of the lateral flow assay (LFA) tests; General procedure for the analysis of samples with the LFA tests; Optimization of the LFA tests; Study of matrix effects; Sensitivity; Specificity. (PDF)

## References

[1] Daguer H., Hoff R. B., Molognoni L., Kleemann C. R., Felizardo L. V. (2018). Outbreaks,
toxicology, and analytical methods of marine toxins in foods. Curr. Opin. Food Chem..

[2] Morabito S., Silvestro S., Faggio C. (2018). How the marine biotoxins
affect human
health. Nat. Prod. Res..

[3] Loeffler C. R., Tartaglione L., Friedemann M., Spielmeyer A., Kappenstein O., Bodi D. (2021). Ciguatera mini review:
21st century
environmental challenges and the interdisciplinary research efforts
rising to meet them. Int. J. Environ. Res. Public
Health.

[4] Perkins J. C., Zenger K. R., Liu Y., Strugnell J. M. (2024). Ciguatera
poisoning: a review of the ecology and detection methods for Gambierdiscus
and Fukuyoa specias. Harmful Algae.

[5] Friedman M. A., Fernandez M., Backer L. C., Dickey R. W., Bernstein J. M., Schrank K., Kibler S. (2017). Un updated review of
ciguatera fish poisoning: clinical, epidemiological, environmental,
and public health management. Mar. Drugs.

[6] Alvito P., Gago-Martínez A. (2025). Ciguatera
toxins, a potential health risk emerging
in Europe: overview of progress and challenges. Curr. Opin. Food Sci..

[7] FAO and WHO . Report of the Expert Meeting on Ciguatera Poisoning. Rome, 19–23 November 2018. Food Safety and Quality 2020, 9. 10.4060/ca8817en.

[8] Holmes M. J., Lewis R. J. (2025). Reviewing evidence for disturbance to coral reefs increasing
the risk of ciguatera. Toxins.

[9] Canals A., Martínez C. V., Diogène J., Gago-Martínez A. (2021). Risk characterization
of ciguatera poisoning in Europe. EFSA Support.
Pub..

[10] Otero P., Silva M. (2022). Emerging marine biotoxins
in European waters: potential risks and
analytical challenges. Mar. Drugs.

[11] Murata M., Legrand A. M., Ishibashi Y., Yasumoto T. (1989). Structures of ciguatoxin
and its congener. J. Am. Chem. Soc..

[12] Inserra, M. ; Lavrukhina, Y. ; Jones, A. ; J. Lewis, R. ; Vetter, I. Ciguatoxin detection methods and high-throughput assays. In Analysis of Food Toxins and Toxicants; 2017 ( Wong, Y.-c. , Lewis, R.J. ). 10.1002/9781118992685.ch15.

[13] Pasinszki T., Lako J., Dennis T. E. (2020). Advances
in detecting ciguatoxins
in fish. Toxins.

[14] Reverté J., Alkassar M., Diogène J., Campàs M. (2023). Detection
of ciguatoxins and tetrodotoxins in seafood with biosensors and other
smart bioanalytical systems. Foods.

[15] Loeffler C. R., Bodi D., Tartaglione L., Dell’Aversano C., Preiss-Weigert A. (2021). Improving in vitro ciguatoxin and
brevetoxin detection:
selecting neuroblastoma (Neuro-2a) cells with lower sensitivity to
ouabain and veratridine (OV-LS). Harmful Algae.

[16] Murata K., Yasumoto T. (2019). Chemiluminescent receptor
binding assay for ciguatoxins
and brevetoxins using acridinium brevetoxin-B2. Toxins.

[17] Yogi K., Oshiro N., Inafuku Y., Hirama M., Yasumoto T. (2011). Detailed LC-MS/MS
analysis of ciguatoxins revealing distinct regional and species characteristics
in fish and causative alga from the Pacific. Anal. Chem..

[18] Hokama Y., Banner A. H., Boylan D. B. (1977). A radioimmunoassay
for the detection
of ciguatoxins. Toxicon.

[19] Tsumuraya T., Takeuchi K., Yamashita S., Fujii I., Hirama M. (2012). Development
of a monoclonal antibody against the left wing of ciguatoxin 1B: thiol
strategy and detection using a sandwich ELISA. Toxicon.

[20] Oguri H., Hirama M., Tsumuraya T., Fujii I., Maruyama M., Uehara H., Nagumo Y. (2003). Synthesis-based approach toward direct
sandwich immunoassay for ciguatoxin CTX3C. J.
Am. Chem. Soc..

[21] Tsumuraya T., Fujii I., Hirama M. (2010). Production of monoclonal antibodies
for sandwich immunoassay detection of Pacific ciguatoxins. Toxicon.

[22] Tsumuraya T., Sato T., Hirama M., Fujii I. (2018). Highly sensitive
and
practical fluorescent sandwich ELISA for ciguatoxins. Anal. Chem..

[23] Leonardo S., Gaiani G., Tsumuraya T., Hirama M., Turquet J., Sagristà N., Rambla-Alegre M., Flores C., Caixach J., Diogène J., O’Sullivan C.
K., Alcaraz C., Campàs M. (2020). Addressing the analytical challenges for the detection
of ciguatoxins using an electrochemical biosensor. Anal. Chem..

[24] Gaiani G., Leonardo S., Tudó À., Toldrà A., Rey M., Andree K. B., Tsumuraya T., Hirama M., Diogène J., O’Sullivan C. K., Alcaraz C., Campàs M. (2020). Rapid detection
of ciguatoxins in Gambierdiscus and Fukuyoa with immunosensing tools. Ecotoxicol. Environ. Saf..

[25] Campàs M., Leonardo S., Oshiro N., Kuniyoshi K., Tsumuraya T., Hirama M., Diogène J. (2022). A smartphone-controlled
amperometric immunosensor for the detection of Pacific ciguatoxins
in fish. Food Chem..

[26] Reverté J., Shukla S., Tsumuraya T., Hirama M., Turquet J., Diogène J., Campàs M. (2025). Analysis of ciguatoxins in fish with
a single-step sandwich immunoassay. Harmful
Algae.

[27] Wang P., Li J., Guo L., Li J., He F., Zhang H., Chi H. (2024). The development of
lateral flow immunochromatographic assay for food
safety in recent 10 years: a review. Chemosensors.

[28] Ji Y., Wang R., Zhao H. (2024). Toward sensitive
and reliable immunoassays
of marine biotoxins: from rational design to food analysis. J. Agric. Food Chem..

[29] Tsumuraya T., Hirama M. (2025). Anti-ciguatoxin monoclonal antibodies:
hapten design,
production, ELISA, and treatment of ciguatera poisoning. Chem. Lett..

[30] Delgado-Corrales B. J., Chopra V., Chauhan G. (2025). Gold nanostars and nanourchins for
enhanced photothermal therapy, bioimaging, and theranostics. J. Mater. Chem. B.

[31] Kamel M., Atta S., Maher S., Abd Elaziz H., Demerdash Z. (2024). Modified streptavidin-biotin based
lateral flow test
strip for rapid detection of SARS-CoV-2 S1 antigen in saliva samples. Sci. Rep..

[32] Calucho E., Parolo C., Rivas L., Álvarez-Diduk R., Merkoçi A. (2020). Nanoparticle-based lateral Flow assays. Compr. Anal. Chem..

[33] Rodríguez M. O., Covián L. B., García A. C., Blanco-López M. C. (2016). Silver
and gold enhancement methods for lateral flow immunoassays. Talanta.

[34] Parolo C., Sena-Torralba A., Bergua J. F., Calucho E., Fuentes-Chust C., Hu L., Rivas L., Álvarez-Diduk R., Nguyen E. P., Cinti S., Quesada-González D., Merkoçi A. (2020). Tutorial:
design and fabrication of nanoparticle-based lateral-flow immunoassays. Nat. Prot..

[35] Oshiro N., Yogi K., Asato S., Sasaki T., Tamanaha K., Hirama M., Yasumoto T., Inafuku Y. (2010). Ciguatera
incidence
and fish toxicity in Okinawa, Japan. Toxicon.

[36] Lehane L., Lewis R. J. (2000). Ciguatera: recent
advances but the risk remains. Int. J. Food
Microbiol..

[37] Oshiro N., Nagasawa H., Nishimura M., Kuniyoshi K., Kobayashi N., Sugita-Konishi Y., Ikehara T., Tachihara K., Yasumoto T. (2023). Analytical studies on ciguateric fish in Okinawa, Japan
(II): the grouper Variola albimarginata. J.
Mar. Sci. Eng..

[38] Oshiro N., Nagasawa H., Watanabe M., Nishimura M., Kuniyoshi K., Kobayashi N., Sugita-Konishi Y., Asakura H., Tachihara K., Yasumoto T. (2022). An extensive survey
of ciguatoxins on grouper Variola louti from the Ryukyu Islands, Japan,
using Liquid Chromatography–Tandem Mass Spectrometry (LC-MS/MS). J. Mar. Sci. Eng..

