# Supplementary material for: Rapid Analysis of Pacific Ciguatoxins in Fish Extracts with a Lateral Flow Assay
Source: Anal Chem. 2026 Jan 9;98(3):1832–8. doi: 10.1021/acs.analchem.5c06159 (PMC12856831; doi:10.1021/acs.analchem.5c06159)
Supplement: Supplementary file 1 [file ac5c06159_si_001.pdf]

SUPPLEMENTARY INFORMATION

Rapid analysis of Pacific ciguatoxins in fish extracts with a lateral flow assay

Ulises G. Díaz-Avello[a,b,#], Vasso Skouridou[a,#,*], Takeshi Tsumuraya[c], Masahiro Hirama[c], Naomasa Oshiro[d], Mònica Campàs[b], Ciara K. O´Sullivan[a,e*]

[a] Interfibio Research Group, Departament d´Enginyeria Química, Universitat Rovira i Virgili, 43007 Tarragona, Spain

[b] Institute of Agrifood Research and Technology (IRTA), 43540 La Ràpita, Spain

[c] Department of Biological Chemistry, Graduate School of Science, Osaka Metropolitan University, Osaka 599-8570, Japan

[d] Division of Biomedical Food Research, National Institute of Health Sciences, Kanagawa 210-9501, Japan

[e] Institució Català de Recerca i Estudis Avancats (ICREA), 08010 Barcelona, Spain

* corresponding authors

ciara.osullivan@urv.cat

vasoula.skouridou@urv.cat

Table of Contents

1. Materials and reagents 3

2. Fish samples and preparation of extracts 3

3. Preparation and assembly of the lateral flow assay (LFA) tests 4

4. General procedure for the analysis of samples with the LFA tests 4

5. Preparation of antibody-nanoparticle conjugates 5

Figure S2. Preparation of the gold nanoparticle conjugates. 6

6. Optimization of the LFA tests 7

6.1. Nanoparticle reporter 7

Figure S3. Choice of nanoparticle reporter for the detection of CTX1B with the LFA tests. 7

6.2. NC membrane type and LFA run time 8

Figure S4. Optimization of the nitrocellulose membrane type and lateral flow assay (LFA) run time. 8

6.3. Test line antibodies 9

Figure S5. Optimization of the concentration of the α-CTXs IgG antibodies (3G8 and 10C9) used to construct the test line on the LFA test membrane. 9

6.4. Running buffer and NC membrane blocking 10

Figure S6. Optimization of the LFA running buffer in the absence of CTXs. 10

7. Study of matrix effects 11

8. Sensitivity 11

Figure S7. Sensitivity of the lateral flow assay (LFA) tests for the detection of CTX1B and 51-OH-CTX3C, individually (200 pg/mL each) or in mixture (400 pg/mL in total). 12

9. Specificity 12

Table S2. Analysis of fish extracts with the LFA tests for the detection of Pacific CTXs. 16

10. References 17

1. Materials and reagents

Toxins and antibodies: CTX1B standard solution was obtained from Prof. Richard J. Lewis (The Queensland University, Australia) and calibrated in relation to the NMR-quantified CTX1B standard solution from Prof. Takeshi Yasumoto (Japan Food Research Laboratories, Japan). 51-OH-CTX3C standard solution was kindly provided by Prof. Takeshi Yasumoto (Japan Food Research Laboratories, Japan) and was used as a model for the series of CTX3C congeners. Saxitoxin (STX) and domoic acid (DA) were from the National Research Council of Canada (NRC, Halifax, Canada), tetrodotoxin (TTX) from Latoxan (France), brevetoxin B (PbTx-2) and okadaic acid (OA) were supplied by Merck (Spain). Antibodies against CTX1B (3G8 IgG), CTX3C (10C9 IgG) or both CTXs (8H4 IgG) were prepared at Osaka Metropolitan University as reported previously.[1,2] The 8H4 IgG was labelled with biotin using the EZ-Link^{TM} Sulfo-NHS LC-LC-Biotin kit (Fisher Scientific, Spain) according to the manufacturer's instructions.

Nanoparticles: the gold nanoparticles (AuNPs; 40 nm diameter at optical density OD 1 in water) and the streptavidin-coated gold nanoparticles (SA@AuNPs; 40 nm diameter at optical density OD 1 in PBS) were provided by BBI Solutions (UK). The gold nanourchins (AuNUs; 90 nm average particle size at OD 1 in 0.1 mM PBS) were from Merck (Spain). The carbon nanoparticles (CNPs; special black 4 powder with average primary particle size of 25 nm) were obtained from Orion Engineered Carbons GmbH (Germany).

Lateral flow assay: the Whatman nitrocellulose (NC) backed membranes (grades FF120HP and FF170HP; height 2.5 cm) and the CF5 wicking pad were purchased from Cytiva (Spain). Fiberglass sample/conjugate release pad grade 8951 was from Ahlström (Finland), backing cards from Kenosha (The Netherlands) and the cassettes from TV Plastics (India).

General reagents: phosphate buffered saline tablets (PBS; 10 mM phosphate buffer pH 7.4, 137 mM NaCl, 2.7 mM KCl), PBST (PBS with 0.05% *v/v* Tween-20), carbonate-bicarbonate buffer (0.2 M, pH 9.4), neutravidin, Tween-20 and boric acid were from Fisher Scientific (Spain). Bovine serum albumin (BSA), goat anti-mouse IgG, streptavidin, skim milk powder, Empigen BB, poly(ethyleneglycol) 20000 (PEG20k), polyvinylpyrrolidone (PVP) and the silver enhancer kit (# SE100) were from Merck (Spain). All other reagents were from Scharlau (Spain), Fisher Scientific (Spain) and Merck (Spain). Milli-Q grade water (18.2 MΩ.cm) was used for all experiments.

2. Fish samples and preparation of extracts

A total of 10 samples from Japan were used for the evaluation of the LFA tests. Of those, 9 were contaminated with CTXs, whereas one (from a *Lutjanus bohar* specimen caught in Okinawa) was not contaminated according to previous LC-MS/MS analysis.[3] The contaminated samples originated from *L. bohar* specimens from Okinawa (*n*=4), *L. bojar* from Wakayama (*n*=2), *Lutjanus monostigma* from Okinawa (*n*=1), *Variola louti* from Okinawa (*n*=1) and *Variola arbimarginata* from Okinawa (*n*=1). A

pool of three non-contaminated *L. bohar* specimens from Okinawa was used for studying matrix effects. The preparation of the extracts was performed at NIHS as detailed previously.[3] Briefly, the fish flesh was extracted twice with acetone, and the combined extracts were evaporated. The remaining aqueous portion was partitioned twice with diethyl ether, and the organic layer was collected and dried. The dried material was dissolved in 90% MeOH, defatted with hexane and the remaining solution was dried. The crude extract was then dissolved in ethyl acetate-MeOH (9:1 *v/v*) and passed through a Florisil cartridge column. The eluate was dried, dissolved in ACN and applied to a primary and secondary amine (PSA) cartridge column. The MeOH eluate was dried under a nitrogen stream. The dried extracts were finally dissolved with PBS at 5000 mg equivalents of fish flesh/mL before analysis with the LFA tests.

3. Preparation and assembly of the lateral flow assay (LFA) tests

To facilitate simultaneous detection of CTX1B and 51-OH-CTX3C, the 3G8 (α-CTX1B) and 10C9 (α-CTX3C) IgG antibodies were combined for the construction of the test line (Figure S1). They were used at final concentrations of 1 or 1.5 mg/mL each in PBS, while a goat anti-mouse IgG antibody was used for the control line at 1 mg/mL in PBS. The antibody solutions were dispensed on the NC membranes using the Automated Lateral Flow Reagent Dispenser (ClaremontBioSolutions LLC, USA) coupled with the Fusion Classic 200 automated syringe pumping system (Chemyx Inc., USA). After dispensing, the membranes were dried for at least 30 min at 37 ºC, and either used directly as is or blocked. For blocking, the membranes were incubated for 30 min with 1% w/v skim milk and 0.5% v/v Empigen BB in 10 mM carbonate-bicarbonate buffer pH 9.4 under mild agitation, rinsed twice with PBS for 10 min and finally dried for 30 min at 37 ºC. For the conjugate release pad (Ahlström grade 8951), this was first briefly immersed in a solution of 1% w/v BSA and 0.05% v/v Tween-20 in 5 mM sodium borate buffer pH 8.8 (pre-treatment) and then dried for 1 h at 37 ºC. The 8H4@AuNPs conjugate at OD 5 in conjugate buffer was then evenly spread on the pre-treated conjugate pad, followed by drying for 1 h at 37 ºC. The sample pad (Ahlström grade 8951) was also pre-treated with 1% w/v BSA in PBST as described above before use. For the final assembly of the strips, the three pads (absorbent, conjugate and sample) were combined with the NC membranes on the adhesive backing cards ensuring a minimum of 2 mm overlap between each pad. The strips were cut at 4 mm using the AUTOKUN HGS201 guillotine cutter from Hangzhou Autokun Technology Co., Ltd. (China), inserted into plastic housing cassettes and stored at 4 ºC in vacuum-sealed bags until use. The intensity of the test lines was quantified using the CubePlus lateral flow reader from opTricon GmbH (Germany).

4. General procedure for the analysis of samples with the LFA tests

The design of the LFA for Pacific CTXs detection is illustrated in Figure S1. For analysis, 50 µL of sample prepared in PBS were applied to the sample window of the LFA test, followed by the addition

of 100 µL of running buffer. The standards were prepared by diluting concentrated MeOH stocks in PBS, whereas the fish MeOH extracts were first evaporated as detailed earlier, followed by resuspension to 5000 mg fish flesh equivalents/mL with PBS. After 20 – 30 min of run time, the results were evaluated by imaging the test with a smartphone and recording the intensity of the test line using the CubePlus LFA reader. Where indicated, a silver enhancer was used to increase the colour intensity of the lines. After running the test, 50 µL of the silver enhancement solution, prepared according to the manufacturer's instructions, were directly dispensed on the test's sample window and the results were recorded after 2 min as detailed above.

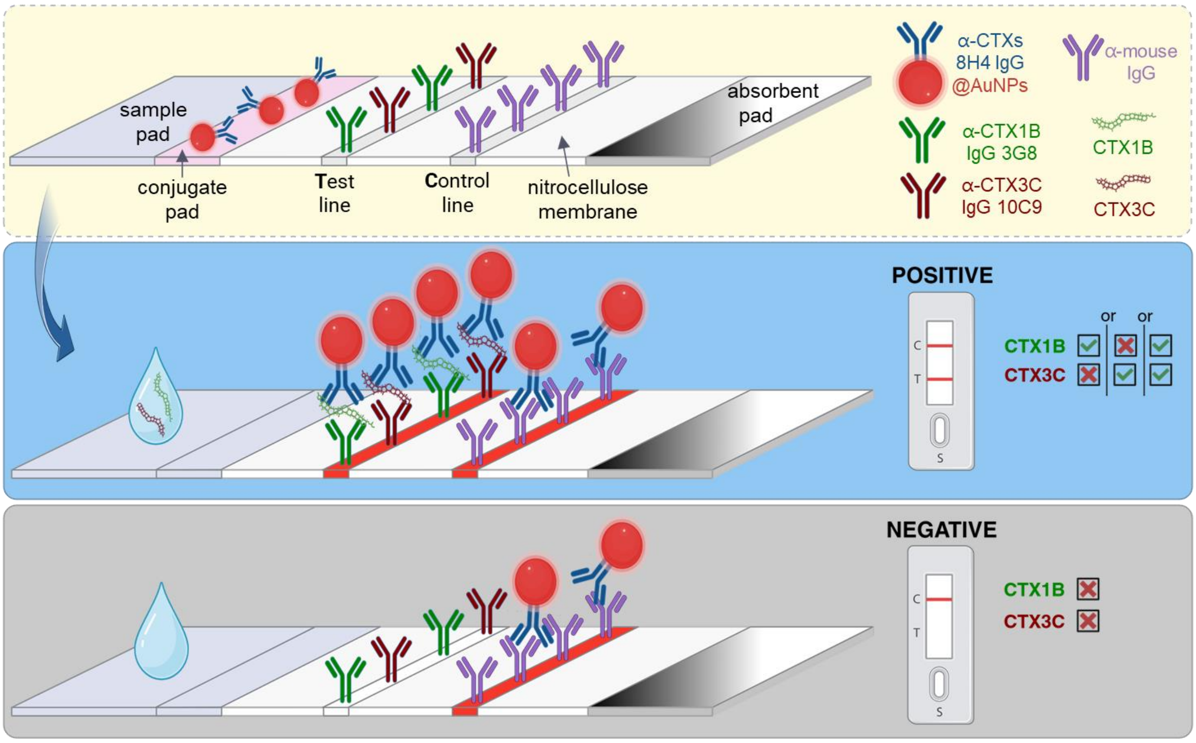

Figure S1. Design of the lateral flow assay (LFA) test for the detection of Pacific ciguatoxins CTX1B and CTX3C and their congeners in fish.

5. Preparation of antibody-nanoparticle conjugates

The concentrations of the antibodies used for the preparation of the gold nanoparticle conjugates were first optimized using a gold aggregation test (Figure S2a) according to a previous report,[4] whereas for the CNPs it was based on a previous report.[5] The 40 nm AuNPs and 90 nm AuNUs suspensions were prepared at OD 1 in water, the 40 nm SA@AuNPs at OD 1 in PBS and the CNPs at 0.2% w/v in 5 mM sodium borate buffer pH 8.8. The particles were initially sonicated for 5 min and then mixed with 8H4 or biotin-8H4 antibody: 4 µg/mL of 8H4 were used for the 8H4@AuNPs, 8 µg/mL of bt-8H4 for the bt-8H4/SA@AuNPs and 100 µg/mL of 8H4 for the 8H4@AuNUs and 8H4@CNPs. The suspensions were

incubated for 30 min (AuNPs and SA@AuNPs) or 1.5 h (CNPs) at 22 ºC under mild agitation, followed by the addition of 1% w/v BSA for blocking and incubation for another 30 min. The conjugates were washed three times by centrifugation for 30 min at 14,000 rpm (10,000 rpm for the CNPs) at 10 ºC and resuspension with the conjugate buffer (5 mM sodium borate buffer pH 9, 1% w/v BSA, 10% w/v sucrose) buffer each time. The final conjugates were resuspended with the same buffer and stored at 4 ºC until further use. The AuNPs and AuNUs conjugates were characterized by UV-Vis spectroscopy (Figure S2b).

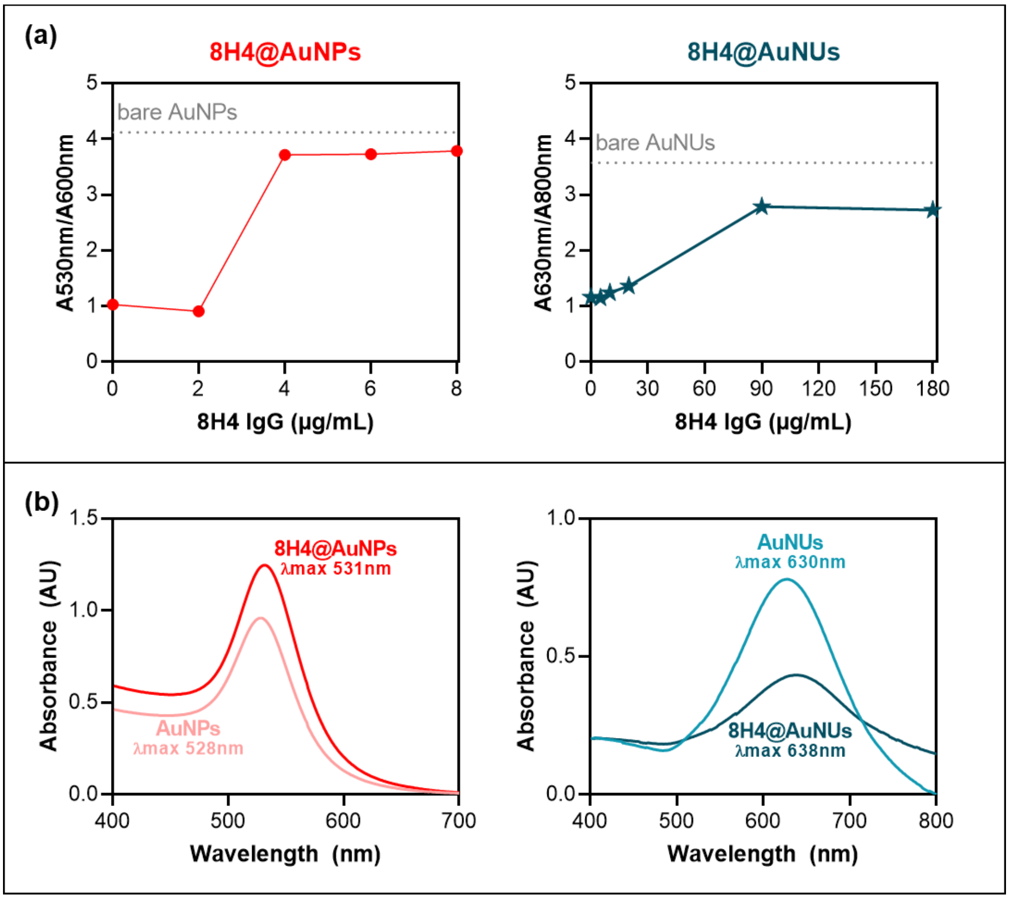

Figure S2. Preparation of the gold nanoparticle conjugates.

(a) Optimization of the concentration of the α-CTXs 8H4 IgG antibody used for the preparation of the gold nanoparticle (AuNPs) and gold nanourchin (AuNUs) conjugates with a gold aggregation test. (b) Absorbance spectra of the 8H4 IgG conjugates with AuNPs and AuNUs.

6. Optimization of the LFA tests

6.1. Nanoparticle reporter

Different 8H4 IgG-nanoparticle conjugates were compared in LFA to choose the conjugate resulting in specific signals with the highest signal-to-noise ratio. Each conjugate was prepared and deposited on the pre-treated conjugate release pads as detailed earlier. The gold nanoparticle-based conjugates (8H4@AuNPs and bt-8H4/SA@AuNPs) were used at OD 5, the gold nanourchins (8H4@AuNUs) at OD 1.5 and the carbon nanoparticles (8H4@CNPs) at 0.02% w/v. Different solutions of the CTX1B were prepared in PBS (0, 40, 200 and 1000 pg/mL) and analyzed using PBS as the running buffer as described above and the results were documented after 20 min of running time (Figure S3).

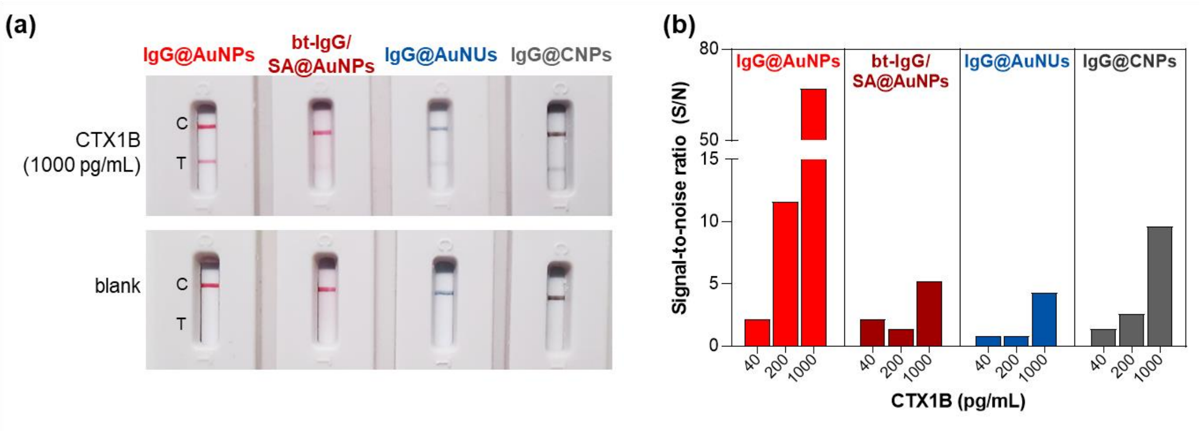

Figure S3. Choice of nanoparticle reporter for the detection of CTX1B with the LFA tests.

The α-CTXs IgG (8H4) was immobilized on bare (AuNPs) or streptavidin-coated gold nanoparticles (SA@AuNPs), gold nanourchins (AuNUs) or carbon nanoparticles (CNPs). Biotinylated IgG was used for preparation of the IgG/SA@AuNPs reporter. The α-CTXs IgG antibodies (3G8 and 10C9) were used at 1.5 mg/mL each. (a) Visual inspection of LFA tests after analysis of CTX1B using the different nanoparticle reporter conjugates. (b) Signal-to-noise (S/N) ratio of LFA tests employing different nanoparticle reporters for the analysis of CTX1B. The test line intensities were measured with the CubePlus LFA reader.

6.2. NC membrane type and LFA run time

Two NC membranes with different flow rates were compared, the FF120HP (90 – 150 sec/4 cm) and FF170HP (140 – 200 sec/4 cm). The tests were carried out using 1000 pg/mL CTX1B in PBS along with a blank control (only PBS) as detailed earlier. The test line intensities were recorded with the LFA reader at different time intervals (Figure S4).

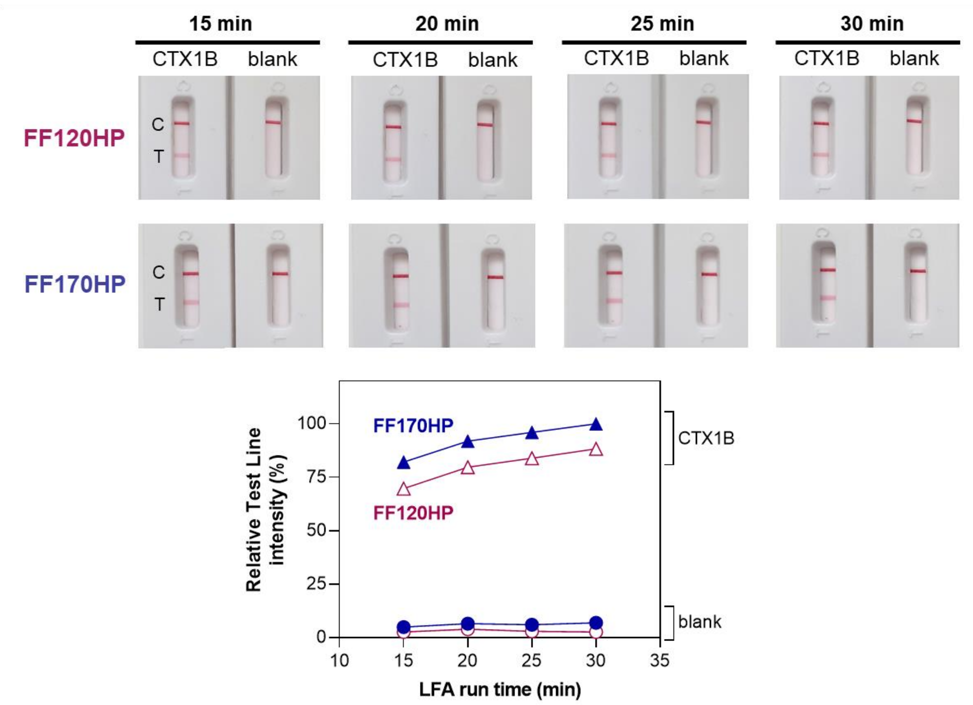

Figure S4. Optimization of the nitrocellulose membrane type and lateral flow assay (LFA) run time.

The α-CTXs test line antibodies (3G8 and 10C9) were used at 1.5 mg/mL each and CTX1B at 1000 pg/mL. Test line intensities were normalized to the % of highest signal in the dataset.

6.3. Test line antibodies

The concentration of the α-CTXs IgG antibodies 3G8 (CTX1B) and 10C9 (CTX3C) used to construct the test line was tested at 1 or 1.5 mg/mL each. The strips were prepared as described earlier and the CTX1B was tested at 600 pg/mL together with a blank sample (only PBS). The intensity of the test lines was recorded after 20 min and 30 min of run time, as well as after the use of the silver enhancer (Figure S5).

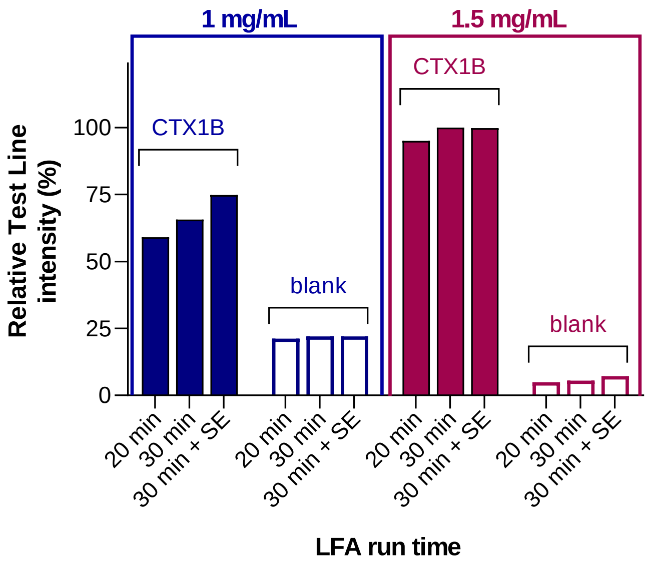

Figure S5. Optimization of the concentration of the α-CTXs IgG antibodies (3G8 and 10C9) used to construct the test line on the LFA test membrane.

SE: silver enhancement. Test line intensities were normalized to the % of highest signal in the dataset.

6.4. Running buffer and NC membrane blocking

To prevent false-positive signals resulting from non-specific binding of the reporter AuNPs to the LFA test line in the absence of CTXs, various blocking agents were initially screened as additives to the running buffer. Blank tests (no-CTXs) were conducted using unblocked NC membranes with PBS as the running buffer, supplemented with one of the following additives: Tween-20 (1% v/v), PEG20k (1% w/v), PVP (1% v/v), BSA (1% w/v), or skim milk (0.1% w/v) (Figure S6A). The additive yielding the lowest non-specific signal at the test line was subsequently tested with both unblocked and blocked membranes, again in the absence of CTXs (Figure S6B), to identify the most suitable conditions for achieving reliable results without background signals.

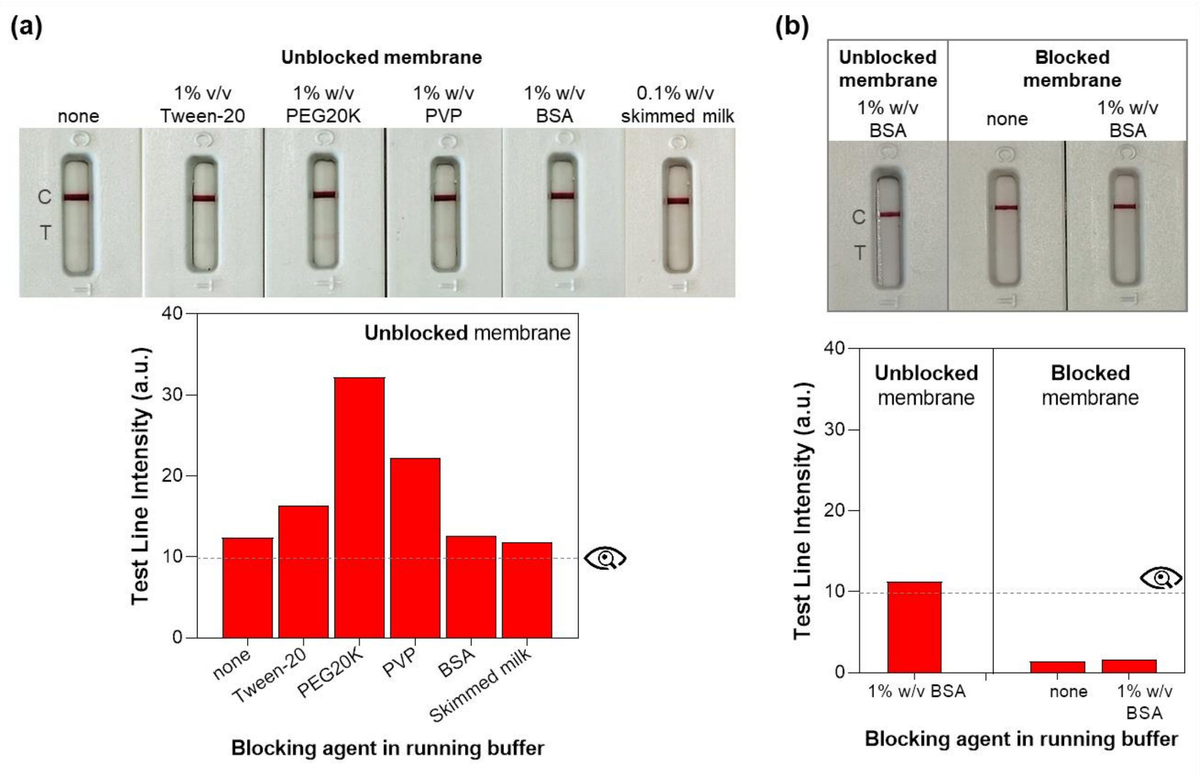

Figure S6. Optimization of the LFA running buffer in the absence of CTXs.

(a) Screening of various blocking agents supplemented to the running buffer (PBS) using unblocked membrane to fabricate the LFA tests. (b) Comparison of unblocked with blocked membrane. The horizontal dashed lines and the eye icons indicate the visual limit of detection, below which no signal is discernible by naked eye inspection.

7. Study of matrix effects

To study the potential fish matrix effects on the LFA tests and calculate the recovery of the CTXs, a pooled negative sample was used. MeOH extracts from three non-contaminated *L. bohar* specimens (Okinawa, Japan) according to LC-MS/MS analysis prepared as detailed earlier were mixed at equal volumes, followed by evaporation and resuspension with PBS at 5000 or 2500 mg fish flesh equivalents/mL. In initial experiments using unblocked LFA strips, CTX1B was spiked at 600 pg/mL in undiluted (5000 mg/mL) or two-fold diluted (2500 mg/mL) fish extract and analyzed as described earlier using 1% w/v BSA in PBS as the running buffer. The results were recorded after 30 min of assay run time. When blocked LFA strips were used, each toxin CTX1B or 51-OH-CTX3C were spiked independently at 1000 pg/mL in undiluted extract (5000 mg/mL) and analyzed as detailed above. In all cases, CTX1B and 51-OH-CTX3C solutions were prepared at the indicated concentrations in PBS and analyzed in parallel for calculating the recovery of the CTXs in the different matrix suspensions. Each sample was analyzed in duplicate.

8. Sensitivity

Solutions of different concentrations of CTX1B and 51-OH-CTX3C (0, 50, 100, 200, 400, 600, 800, 1000 and 2000 pg/mL in PBS) were prepared and analyzed with the LFA tests as detailed earlier. The running buffer was 1% w/v BSA in PBS and the results were recorded after 30 min. For calculation of the limit of detection (LOD), the intensities of the test lines measured with the LFA reader were plotted against the logarithm of CTX concentration and the data were fitted to the four-parameter logistic model (4PL) of GraphPad Prism 8. The LODs were interpolated from the fitted curves as the bottom of the curves plus three times their standard error. Each CTX standard solution was analyzed in duplicate. To further demonstrate the sensitivity of the assay, we tested each CTX individually at a concentration below the LOD, as well as a mixture in which the combined concentration corresponded to the vLOD. While the individual toxins at 200 pg/mL produced no visible test lines, their 200 + 200 pg/mL mixture resulted in a detectable positive signal (Figure S7).

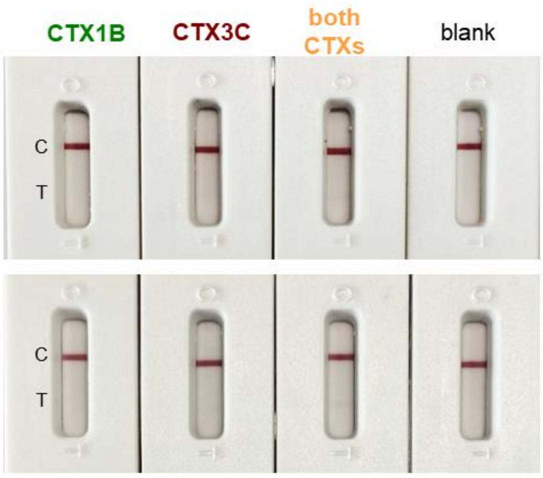

Figure S7. Sensitivity of the lateral flow assay (LFA) tests for the detection of CTX1B and 51-OH-CTX3C, individually (200 pg/mL each) or in mixture (400 pg/mL in total).

9. Specificity

To evaluate the specificity of the LFA test, several marine toxins were analyzed either alone or in a mixture with each of the CTXs. Specifically, CTX1B, 51-OH-CTX3C, tetrodotoxin (TTX), saxitoxin (STX), brevetoxin B (PbTx-2), okadaic acid (OA) and domoic acid (DA) were analyzed at 1000 pg/mL each, whereas individual mixtures of CTX1B or 51-OH-CTX3C with each one of the other toxins (1000 pg/mL each) were also employed to study the potential inference of the other toxins with CTXs detection. Each sample was analyzed in duplicate as detailed earlier using 1% w/v BSA in PBS as the running buffer, while the results were evaluated after 30 min.

Table S1. Lateral flow assays for marine toxins reported in the literature.

T-Prot: toxin-protein conjugate; QD-Prot: quantum dots-protein conjugate; IgG: toxin antibody; IgG@AuNPs: toxin antibody-gold nanoparticle conjugate; IgG@AuNFs: toxin antibody-gold nanoflower conjugate; IgG@FLMs: toxin antibody-fluorescent microspheres conjugate; IgG@LMs: toxin antibody-colored latex microspheres conjugate; Ab/Au@Pt: anti-species antibody conjugated to core-shell gold-platinum nanoparticles; Ab@AuNPs: anti-species antibody conjugated to gold nanoparticles; IgG@HNM: toxin IgG-hollow nanogold microspheres conjugate; btIgG: biotinylated toxin antibody; Neu@AuNPs: neutravidin-gold nanoparticles conjugate; TTX: tetrodotoxin; OA: okadaic acid; DA: domoic acid; STXs: saxitoxins; PbTx-2: brevetoxin B; CIs: cyclic imines; DTXs: dinophysistoxins; MC-LR: microcystin-LR; LOD: limit of detection; IC50: half-maximal inhibitory concentration; N/A: not available.

**SINGLE ANALYTE**								
**Toxin**	**Type**	**Test line**	**Reporter**	**Time**	**LOD (ng/mL)**	**Range (ng/mL)**	**Samples**	**Ref.**
TTX	Signal-off, competitive, colorimetric	T-Prot	IgG@AuNPs	10 min	40	40 – 8000	Pufferfish (spiked)	6
	Signal-off, competitive, colorimetric	T-Prot	IgG@AuNPs	5 min	2 mg/kg	N/A	Pufferfish (contaminated)	7
	Signal-off, competitive, colorimetric	T-Prot	IgG@AuNPs	10 min	20	20 – 80	Clams (spiked)	8
	Signal-off, competitive, colorimetric	T-Prot	IgG@AuNPs	12 min	0.78	0.78 – 50	Pufferfish (spiked)	9
	Signal-on, competitive, fluorescent	QD-Prot<br>T-Prot	IgG@AuNFs	8 min	0.2	1.56 – 100	Pufferfish (spiked)	10
	Signal-off, competitive, colorimetric	T-Prot	IgG@AuNPs	5 min	10	1 – 50	Crucian and clam (spiked)	11
	Signal-on, competitive, fluorescent	T-Prot	IgG@FLMs	20 min	0.05	0.5 – 40	Sea flower beetle and clams (spiked)	12
	Signal-off, competitive, colorimetric	T-Prot	IgG@AuNFs<br>IgG@LMs	10 min	5.4<br>9.5	9.5 - 331<br>5.4 – 443	Yellow croaker, grass carp, perch, pufferfish (spiked)	13

	Signal-on, sandwich, colorimetric	ssDNA Aptamer	IgG@AuNPs	20 min	0.31	0.31 – 10	Pufferfish (contaminated)	14
	Signal-on, sandwich, colorimetric	IgG	ssDNA aptamer@AuNPs	15 min	8	8 – 100	Pufferfish (spiked)	15
OA	Signal-off, competitive, colorimetric	T-Prot	IgG@AuNPs	10 min	10	10 – 50	Scallops, clams, sea snails, oysters (spiked)	16
	Signal-off, competitive, colorimetric	T-Prot	IgG and Ab/Au@Pt	20 min	0.5	0.8 – 6.8	Seawater and trout (spiked)	17
DA	Signal-off, competitive, colorimetric	T-Prot	IgG@AuNPs s	N/A	0.5	0.25 –50	Mussels, oysters, scallops, clams, cockles (spiked)	18
	Signal-off, competitive, colorimetric	T-Prot	IgG and Ab@AuNPs	15 min	1.4	2.9 – 35.8	Seawater, mussels, shrimps, octopuses (spiked)	19
STXs	Signal-off, competitive, colorimetric	T-Prot	IgG@AuNPs	5 min	0.1	N/A	Mussels, oysters, scallops, clams, cockles (contaminated)	20
	Signal-off, competitive, colorimetric	T-Prot	IgG@AuNPs	N/A	0.049 (IC50)	0.018 – 3.9	Seawater, mussels, sea snails (spiked)	21
PbTx-2	Signal-on, competitive, colorimetric	T-Prot	IgG@HNM	10 min	0.1	0.05 – 0.1	Mussels and clams (spiked)	22
CIs	Signal-on, competitive, colorimetric	Receptor	btIgG and Neu@AuNPs	N/A	1.03 µM (IC50)	N/A	Mussels and cockles (contaminated)	23
CTXs	Signal-on, sandwich, colorimetric	IgGs	IgG@AuNPs	20 min	0.4 (vLOD)<br>0.3 – 0.4 (calculated)<br>0.1 µg/kg	0.4 – 2	Snapper, grouper (contaminated)	This work

MULTIPLE ANALYTES								
**Toxin**	**Type**	**Test line**	**Reporter**	**Time**	**LOD (ng/mL)**	**Range (ng/mL)**	**Samples**	**Ref.**
TTX<br>OA	Signal-off, competitive, colorimetric	T-Prot	IgG@AuNPs	10 min	15	1 – 20	Clams (contaminated)	24
OA<br>DTXs	Signal-off, competitive, colorimetric	T-Prot	IgG@AuNPs	10 min	N/A	N/A	Variety of seafood (contaminated)	25
OA<br>DTXs	Signal-off, competitive, colorimetric	T-Prot	IgG@AuNPs	> 1 h	0.030	0.01 – 1	Seawater, trout, shrimps, scallops (spiked)	26
OA<br>DTXs	Signal-off, competitive, colorimetric	T-Prot	IgG@AuNPs	15 min	N/A	0.5 – 10	Mussels, oysters, clams (contaminated)	27
OA<br>DA	Signal-off, competitive, colorimetric	T-Prot	IgG and Ab@AuNPs	18 min	0.1 (OA)<br>1.2 (DA)	N/A	Seawater, mussels, shrimps, scallops, octopuses, whelks, crabs (spiked)	28
OA<br>DA<br>STXs	Signal-off, competitive, colorimetric	T-Prot	IgG@AuNPs	35 min	0.1 (OA)<br>4.4 (DA)<br>1.1 (STX)	0.2 – 1.5 (OA)<br>8.2 – 140.3 (DA)<br>2.5 – 65 (STX)	Mussels (spiked)	29
OA<br>DA<br>MC-LR	Signal-off, competitive, colorimetric	T-Prot	IgG and Ab@AuNPs	18 min	0.1 (OA)<br>1.3 (DA)<br>0.05 (MC-LR)	0.2 – 1.1 (OA)<br>3.2 – 58.2 (DA)<br>0.07 – 0.29 (MC-LR)	Seawater and trout (spiked)	30

Table S2. Analysis of fish extracts with the LFA tests for the detection of Pacific CTXs.

The extracts were analyzed directly at 5000 mg flesh eq./mL (without dilution), except for FE20111 (1667 mg flesh eq./mL; dilution 1/3) and FE20112 (2500 mg flesh eq./mL; dilution 1/2). The total CTXs were quantified by LC-MS/MS at 5000 mg flesh eq./mL. The results of the LFA by visual inspection are scored as negative (-), weak positive (+), positive (++) and strong positive (++). Semi-quantification of the LFA results was performed with the Cube LFA reader (*n*=2). LC-MS/MS LOD 0.002 µg/kg (0.01 ng/mL), LOQ 0.005 µg/kg (0.025 ng/mL); LFA visual LOD: 0.4 ng/mL; LFA LOD: 0.3 – 0.4 ng/mL.

**Sample ID**	**Species**	**Origin**	**LC-MS/MS (µg/kg)**						**LC-MS/MS (pg/mL)**	**LFA**	
			**CTX1B**	**52-*epi*-54-deoxyCTX1B**	**54-deoxy CTX1B**	**2,3,51-trihydroxy CTX3C**	**2,3-dihydroxy CTX3C**	**Total CTXs**	**Total CTXs**	**Visual**	**Total CTXs (pg/mL)**
NIHS-FE20101	*Lutjanus bohar*	Okinawa	0.029	0.028	0.038	< LOD	< LOD	0.095	478	+	349
NIHS-FE20102	*Lutjanus bohar*	Wakayama	0.103	0.012	0.019	0.008	0.009	0.151	755	+++	3725
NIHS-FE20103	*Lutjanus bohar*	Wakayama	0.099	0.010	0.020	0.008	0.007	0.144	727	+++	> 3800
NIHS-FE20107	*Lutjanus bohar*	Okinawa	0.040	0.024	0.057	< LOD	< LOD	0.121	608	++	532
NIHS-FE20110	*Lutjanus monostigma*	Okinawa	0.089	0.013	0.009	< LOD	< LOD	0.111	552	+++	2322
NIHS-FE20111	*Variola louti*	Okinawa	0.105	0.054	0.156	< LOD	< LOD	0.315	1574	++	2019
NIHS-FE20112	*Variola albimarginata*	Okinawa	0.067	0.087	0.093	< LOD	< LOD	0.247	1237	+	846
NIHS-FE24001	*Lutjanus bohar*	Okinawa	< LOD	< LOD	< LOD	< LOD	< LOD	< LOD	< LOD	-	< LOD
NIHS-FE24002	*Lutjanus bohar*	Okinawa	0.019	0.024	0.066	< LOD	< LOD	0.109	543	-	274
NIHS-FE24003	*Lutjanus bohar*	Okinawa	0.013	< LOQ	< LOQ	< LOD	< LOD	0.013	66	-	< LOD

10. References

1. Tsumuraya, T.; Fujii, I.; Hirama, M. Toxicon 2010, 56, 797–803. https://doi.org/10.1016/j.toxicon.2009.06.003
2. Tsumuraya, T.; Takeuchi, K.; Yamashita, S.; Fujii, I.; Hirama, M. Toxicon 2012, 60, 348 – 357. https://doi.org/10.1016/j.toxicon.2012.04.34
3. Campàs, M.; Leonardo, S.; Oshiro, N.; Kuniyoshi, K.; Tsumuraya, T.; Hirama, M.; Diogène, J. Food Chem. 2022, 374, 131687. https://doi.org/10.1016/j.foodchem.2021.131687
4. Díaz-Avello, U.; Skouridou, V.; Shkembi, X.; Reverté, J.; Mandalakis, M.; Peristeraki, P.; Campàs, M.; O'Sullivan, C. K. Sci. Total Environ. 2025, 978, 179419. https://doi.org/10.1016/j.scitotenv.2025.179419
5. Ginés, I.; Gaiani, G.; Ruhela, A.; Skouridou, V.; Campàs, M.; Masip, L. Harmful Algae 2021, 110, 102135. https://doi.org/10.1016/j.hal.2021.102135
6. Zhou, Y.; Li, Y.; Lu, S.; Ren, H.; Li, Z.; Zhang, Y.; Pan, F.; Liu, W.; Zhang, J.; Liu, Z. Gold nanoparticle probe-based immunoassay as a new tool for tetrodotoxin detection in puffer fish tissues. Sens. Actuators B Chem. 2010, 146, 368-372. https://doi.org/10.1016/j.snb.2010.02.049
7. Thattiyaphong, A.; Unahalekhaka, J.; Mekha, N.; Nispa, W.; Kluengklangdon, P.; Rojanapantip, L. Efficiency of a rapid test for detection of tetrodotoxin in puffer fish. J. Immunoassay Immunochem. 2013, 35, 111-119. https://doi.org/10.1080/15321819.2013.802698
8. Ling, S.; Chen, Q. A.; Zhang, Y.; Wang, R.; Jin, N.; Pang, J.; Wang, S. Development of ELISA and colloidal gold immunoassay for tetrodotoxin detection based on monoclonal antibody. Biosens. Bioelectron. 2015, 71, 256-260. https://doi.org/10.1016/j.bios.2015.04.049
9. Shen, H.; Zhang, S.; Fu, Q.; Xiao, W.; Wang, S.; Yu, S.; Xiao, M.; Bian, H.; Tang, Y. A membrane-based fluorescence-quenching immunochromatographic sensor for the rapid detection of tetrodotoxin. Food Control 2017, 81, 101-106. https://doi.org/ 10.1016/j.foodcont.2017.06.001
10. Shen, H.; Xu, F.; Xiao, M.; Fu, Q.; Cheng, Z.; Zhang, S.; Huang, C.; Tang, Y. A new lateral-flow immunochromatographic strip combined with quantum dot nanobeads and gold nanoflowers for rapid detection of Tetrodotoxin. Analyst 2017, 142, 4393-4398. https://doi.org/10.1039/c7an01227f
11. Li, Y.; Xu, X.; Liu, L.; Kuang, H.; Xu, L.; Xu, C. A gold nanoparticle-based lateral flow immunosensor for ultrasensitive detection of tetrodotoxin. Analyst 2020, 145, 2143. https://doi.org/10.1039/d0an00170h
12. Sun, J.; Shi, Z.; Zhang, T.; Wang, L.; Dong, R.; Zhang, Y.; Sun, X. Highly sensitive and quantitative fluorescent strip immunosensor based on an independent control system for rapid detection of tetrodotoxin in shellfish. Food Control 2023, 145, 109403. https://doi.org/10.1016/j.foodcont.2022.109403
13. Huang, Y.; Xu, A.; Xu, Y.; Wu, H.; Sun, M.; Madushika, L.; Wang, R.; Yuan, J.; Wang, S.; Ling, S. Sensitive and rapid detection of tetrodotoxin based on gold nanoflower- and latex microsphere-labeled monoclonal antibodies. Front. Bioeng. Biotechnol. 2023, 11, 1196043. https://doi.org/10.3389/fbioe.2023.1196043
14. Díaz-Avello, U. G.; Skouridou, V.; Shkembi, X.; Reverté, J.; Mandalakis, M.; Peristeraki, P.; Campàs, M.; O'Sullivan, C. K. Aptamer-antibody sandwich lateral flow test for rapid visual detection of tetrodotoxin in pufferfish. Sci. Total Environ. 2025, 978, 179419. https://doi.org/10.1016/j.scitotenv.2025.179419
15. Tang, Y.; Yao, L.; Wang, Y.; Lin, B.; Yao, Y.; Chen, L.; Huang, H.; Xu, J.; Guo, L. Signal-on lateral flow immunoassays for rapid detection of tetrodotoxin in pufferfish. J. Hazar. Mater. 2025, 486, 136973. https://doi.org/10.1016/j.jhazmat.2024.136973
16. Lu, S. -Y.; Lin, C.; Li, Y. -S.; Zhou, Y.; Meng, X. -M.; Yu, S. -Y.; Li, Z. -H.; Li, L.; Ren, H. -R.; Liu, Z. -S. A screening lateral flow immunochromatographic assay for on-site detection of okadaic acid in shellfish products. Anal. Biochem. 2012, 422, 59–65. http://doi.org/10.1016/j.ab.2011.12.039

17. Hendrickson, O. D.; Zvereva, E. A.; Panferov, V. G.; Solopova, O. N.; Zherdev, A. V.; Sveshnikov, P. G.; Dzantiev, B. B. Application of Au@PT nanozyme as enhancing label for the sensitive lateral flow immunoassay of okadaic acid. Biosensors 2022, 12, 1137. https://doi.org/10.3390/bios12121137

18. Jawaid, W.; Meneely, J.; Campbell, K.; Hooper, M.; Melville, K.; Holmes, S.; Rice, J.; Elliot, C. T. Development and validation of the first high performance-lateral flow immunoassay (HP-LFIA) for the rapid screening of domoic acid from shellfish extracts. Talanta 2013, 116, 663–669. http://doi.org/10.1016/j.talanta.2013.07.027

19. Hendrickson, O. D.; Zvereva, E. A.; Solopova, O. N.; Varlamov, N. E., Shemchukova, O. B.; Zherdev, A. V.; Sveshnikov, P. G.; Dzantiev, B. B. Rapid detection of phycotoxin domoic acid in seawater and seafood based on the developed lateral flow immunoassay. Anal. Methods 2022, 14, 2446. https://doi.org/10.1039/d2ay00751g

20. Jawaid, W.; Campbell, K.; Melville, K.; Holmes, S. J.; Rice, J.; Elliot, C. T. Development and validation of a novel lateral flow immunoassay (LFIA) for the rapid screening of paralytic shellfish toxins (PSTs) from shellfish extracts. Anal. Chem. 2015, 87, 5324–5332. https://doi.org/10.1021/acs.analchem.5b00608

21. Wei, L. -N.; Luo, L.; Yin, Q. -Y.; Lei, H.; Wang, B. -Z.; Guan, T.; Xu, Z. -L. Ultrasensitive "hunter of target": rabbit monoclonal antibody-based competitive lateral flow immunoassay for saxitoxin. Anal. Chem. 2025, 97, 1410–1418. https://doi.org/10.1021/acs.analchem.4c05998

22. Zhang, K.; Wu, J.; Li, Y.; Wu, Y.; Huang, T.; Tang, D. Hollow nanogold microsphere-signalized lateral flow immunodipstick for the sensitive determination of the neurotoxin brevetoxin B. Microchim. Acta 2014, 181, 1447–1454. https://doi.org/10.1007/s00604-014-1291-9

23. Noirmain, F.; Dano, J.; Hue, N.; Gonzalez-Jartin, J. M.; Botana, L. M.; Servent, D.; Simon, S.; Aráoz, R. NeuroTorp, a lateral Flow test based on toxin-receptor affinity for in-situ early detection of cyclic imine toxins. Anal. Chim. Acta 2022, 1221, 339941. https://doi.org/10.1016/j.aca.2022.339941

24. Ling, S.; Li, X.; Zhang, D.; Wang, K.; Zhao, W.; Zhao, Q.; Wang, R.; Yuan, J.; Xin, S.; Wang, S. Detection of okadaic acid (OA) and tetrodotoxin (TTX) simultaneously in seafood samples using colloidal gold immunoassay. Toxicon 2019, 165, 103–109. https://doi.org/10.1016/j.toxicon.2019.04.011

25. Lin, C.; Liu, Z. -S.; Tan, C. -Y.; Guo, Y. -P.; Li, L.; Ren, H. -L.; Li, Y. -S.; Hu, P.; Gong, S.; Zhou, Y.; Lu, S. -Y. Contamination of commercially available seafood by key diarrhetic shellfish poisons along the coast of China. Environ. Sci. Pollut. Res. 2015, 22, 1545–1553. https://doi.org/10.1007/s11356-014-3494-3

26. Hendrickson, O. D.; Zvereva, E. A.; Zherdev, A. V.; Dzantiev, B. B. Cascade-enhanced lateral flow immunoassay for sensitive detection of okadaic acid in seawater, fish, and seafood. Foods 2022, 11, 1691. https://doi.org/10.3390/foods11121691

27. Jawaid, W.; Meneely, J. P.; Campbell, K.; Melville, K.; Holmes, S. J.; Rice, J.; Elliot, C. T. Development and validation of a lateral flow immunoassay for the rapid screening of okadaic acid all dinophysis toxins from shellfish extracts. J. Agric. Food Chem. 2015, 63, 8574–8583. https://doi.org/10.1021/acs.jafc.5b01254

28. Hendrickson, O. D.; Zvereva, E. A.; Solopova, O. N.; Zherdev, A. V.; Sveshnikov, P. G.; Eremin, S. A.; Dzantiev, B. B. Double immunochromatographic test system for sensitive detection of phycotoxins domoic acid and okadaic acid in seawater and seafood. Micromachines 2022, 13, 1506. https://doi.org/10.3390/mi13091506

29. Mills, C.; Dillon, M. J.; Kulabhusan, P. K.; Senovilla-Herrero, D.; Campbell, K. Multiplex lateral flow assay and the sample preparation method for the simultaneous detection of three marine toxins. Environ. Sci. Technol. 2022, 56, 12210–12217. https://doi.org/10.1021/acs.est.2c02339

30. Zvereva, E. A.; Hendrickson, O. D.; Solopova, O. N.; Zherdev, A. V.; Sveshnikov, P. G.; Dzantiev, B. B. Triple immunogromatographic test system for detection of priority acquatic toxins in water and fish. Anal. Bioanal. Chem. 2022, 414, 7553–7563. https://doi.org/10.1007/s00216-022-04298-8.
